# The extraction of risk factor and its application for elderly patients with dementia based on accidental cases

**DOI:** 10.1002/alz70861_108271

**Published:** 2025-12-23

**Authors:** Seok Woo Moon, Sung Man Chang, Beomjun Kim

**Affiliations:** ^1^ Konkuk University School of Medicine, Chungju, Chungbuk‐do Korea, Republic of (South); ^2^ Kyungpook National University, Daegu, Daegu Korea, Republic of (South); ^3^ Konkuk University Chungju Hospital, Chungju, Chungbuk Korea, Republic of (South)

## Abstract

**Background:**

The aim of this study was first, to extract the risk factor by investigating several cases of accident of older dementia patients at home, and second, based on these results to provide basic information for the determination of monitoring factor for the care of older dementia patients.

**Method:**

Basic and behavioral characteristics, Short form of Samsung Dementia Questionnaire (S‐SDQ), Activities of Daily Living (ADL), and cases of accident were investigated with 55 older dementia patients at home (16 male, 39 female). Based on these questionnaires, risk factors were extracted and frequency, cooccurrence frequency, and occurring place of risk factors, presence or not, region, and degree of injury were investigated. Frequency between risk factors and behavioral characteristics, ADL, and S‐SDQ was analyzed by crosstabulation frequency analysis.

**Result:**

Results showed that several risk factors were extracted, and the frequency of 'going out' was the highest, and risk factors for injury were 'tumble', 'bump', 'slip', and 'fall'. Cooccurrence frequency analysis showed that the occurrence of 'fall', 'going out', 'fire of gas', and 'violence' with other factors was relatively higher than others. The occurring place of risk factor was the highest in home neighborhood, and the region of injury in knee, and the degree of injury with bruise. Crosstabulation frequency analysis showed that factors which had difference in frequency of risk factor were behavioral disorder, disorder of daily living and ADL.

**Conclusion:**

This study presents various basic information necessary for monitoring older dementia patients based on accident cases of the dementia patients.

